# Supplementary material for: The Consumption of Food-Based Iodine in the Immediate Pre-Pregnancy Period in Madrid Is Insufficient. San Carlos and Pregnancy Cohort Study
Source: Nutrients. 2021 Dec 14;13(12):4458. doi: 10.3390/nu13124458 (PMC8707458; doi:10.3390/nu13124458)
Supplement: Supplementary file 1 [file nutrients-13-04458-s001.zip › nutrients-1493114-supplementary.pdf]

Table S1 Estimation of Iodine content in food items used for calculation of iodine intake from the adapted DNCT questionnaire.

Food Items in DNCT questionnaire	Specific Food content in a serving	Serving (gr)	Iodine content (μg/100 g) per food group serving	
			Mean + SD	Median (Q1-Q3)
Vegetables<br>1436 serving	Chard (0.05)<br>Onion (0.39)<br>Green beans (0.07)<br>Carrot (0.05)<br>Tomato (0.14)<br>Lettuce (0.25)<br>Spinach (0.05)<br>Pepper (0.08)<br>Zucchini (0.11)<br>Mushroom (0.07)	150	24.1 + 9.4	22.8 (19.5-26.5)
Pieces of fruit<br>(1213 serving)	Oranges (0.20)<br>Apples (0.16)<br>Pears (0.07)<br>Strawberries (0.07)<br>Plums (0.02)<br>Peach (0.02)<br>Banana (0.12)<br>Grapes (0.07)<br>Pineapple (0.06)<br>Kiwi (0.02)<br>Apricot (0.08)<br>Melons (0.11)	160	9.9 + 9.3	7.1 (3.2-14.4)
Nuts<br>(205 serving)	Walnuts (0.15)<br>Hazelnuts (0.13)<br>Almonds (0.18)<br>Pistachio (0.22)<br>Peanut (0.32)	20	2.9 + 4.5	1.9 (0-4.0)
White fish<br>(276 serving)	Hake (0.22)<br>Whiting (0.27)<br>Cod (0.16)<br>Sole (0.09)<br>Bass (0.21)<br>Snuff (0.07)	150	5.5 + 4.3	4.5 (1.1-6.7)
Fatty Fish<br>(227 serving)	Sardine (0.19)<br>Anchovy (0.16)<br>Tuna (0.16)<br>Mackerel (0.11)<br>Trout (0.10)<br>Salmon (0.16)<br>Melva (0.06)<br>Swordfish (0.06)	150	14.3 + 10.5	12.9 (4.5-22.5)
Conservas<br>(273 serving)	Tuna (0.25)<br>Sardines (0.23)<br>Mackerel (0.12)<br>Mussel (0.17)<br>Melva (0.07)<br>Cockles (0.06)<br>Anchovies (0.11)	50	11.2 + 13.5	9.8 (1.6-19.5)
Shellfish	Mussel (0.24)	150	24.2 + 29.9	9.4 (5.2-19.5)

(285 serving)	Prawns (0.30)<br>Clams (0.11)<br>Squid (0.21)<br>Octopus (0.08)<br>Crustacean (0.07)

Foods that did not reach 1% consumption (0.01 of serving) were not taken into account
